# The effects of orally administered *Bacillus coagulans* and inulin on prevention and progression of rheumatoid arthritis in rats

**DOI:** 10.3402/fnr.v60.30876

**Published:** 2016-07-15

**Authors:** Khadijeh Abhari, Seyed Shahram Shekarforoush, Saeid Hosseinzadeh, Saeid Nazifi, Javad Sajedianfard, Mohammad Hadi Eskandari

**Affiliations:** 1 Department of Food Hygiene and Public Health, School of Veterinary Medicine, Shiraz University, Shiraz, Iran; 2 Department of Clinical Study, School of Veterinary Medicine, Shiraz University, Shiraz, Iran; 3 Department of Physiology, School of Veterinary Medicine, Shiraz University, Shiraz, Iran; 4 Department of Food Science and Technology, School of Agriculture, Shiraz University, Shiraz, Iran

**Keywords:** rheumatoid arthritis, *Bacillus coagulans*, inulin, animal model

## Abstract

**Background:**

Probiotics have been considered as an approach to addressing the consequences of different inflammatory disorders. The spore-forming probiotic strain *Bacillus coagulans* has demonstrated anti-inflammatory and immune-modulating effects in both animals and humans. The prebiotic inulin also potentially affects the immune system as a result of the change in the composition or fermentation profile of the gastrointestinal microbiota.

**Objective:**

In the present study, an *in vivo* model was conducted to investigate the possible influences of probiotic *B. coagulans* and prebiotic inulin, both in combination and/or separately, on the downregulation of immune responses and the progression of rheumatoid arthritis (RA), using arthritis-induced rat model.

**Design:**

Forty-eight healthy male Wistar rats were randomly categorized into six experimental groups as follows: 1) control: normal healthy rats fed with standard diet, 2) disease control (RA): arthritis-induced rats fed with standard diet, 3) prebiotic (PRE): RA+ 5% w/w long-chain inulin, 4) probiotic (PRO): RA+ 10^9^ spores/day *B. coagulans* by orogastric gavage, 5) synbiotic (SYN): RA+ 5% w/w long-chain inulin and 10^9^ spores/day *B. coagulans*, and 6) treatment control: (INDO): RA+ 3 mg/kg/day indomethacin by orogastric gavage. Feeding with the listed diets started on day 0 and continued to the end of study. On day 14, rats were injected with complete Freund's adjuvant (CFA) to induce arthritis. Arthritis activity was evaluated by the biochemical parameters and paw thickness. Biochemical assay for fibrinogen (Fn), serum amyloid A (SAA), and TNF-α and alpha-1-acid glycoprotein (α_1_*AGp*) was performed on day 21, 28, and 35 (7, 14 and 21 days post RA induction), respectively.

**Results:**

Pretreatment with PRE, PRO, and SYN diets significantly inhibits SAA and Fn production in arthritic rats (*P* < 0.001). A significant decrease in the production of pro-inflammatory cytokines, such as TNF-α, was seen in the PRE, PRO, and SYN groups (*P* < 0.001), which was similar to the anti-inflammatory effect of indomethacin. Furthermore, no significant anti-inflammatory effects were observed following different treatments using α_1_*AGp* as an RA indicator. Pretreatment with all supplied diets significantly inhibited the development of paw swelling induced by CFA (*P* < 0.001).

**Conclusion:**

The results of this study indicate that the oral intake of probiotic *B. coagulans* and prebiotic inulin can improve the biochemical and clinical parameters of induced RA in rat.

Rheumatoid arthritis (RA) is considered one of the imperative chronic autoimmune, inflammatory diseases of indistinct origin. Evidence suggests that patients with RA have significant differences in intestinal microbiota compared to healthy control patients. RA patients show a significant decrease in *Bifidobacterium* species and lactic acid bacteria (LAB) with various reports of high and low *Clostridium* species (1). Therefore, dietary manipulation through probiotic therapy may be a noninvasive approach regulating the gut microbiota with the aim of maintaining proper gastrointestinal (GI), downregulating the abnormal inflammatory response, and relieving symptoms of RA (2). It is not fully clear how probiotics prevent or treat arthritis; however, a decrease in gut permeability, which was closely associated with the modulation of immune systems, was formerly reported by other researchers. This modulation role may due to increasing local secretory IgA immune responses to pathogens, reducing the overgrowth of pathogenic bacteria, and downregulating inflammatory immune factors, such as IFN-γ, IL-12, and TNF-α, without altering regulatory cytokines, such as IL-10 and TGF-β (3, 4).

Prebiotics are specific substances that encourage the growth of certain bacteria, microorganisms that then contribute to the health of their hosts. One of the most popular prebiotics is inulin, which is extracted from chicory root. Synbiotics are functional foods, a mixture of probiotics and prebiotics intended to increase the survival and colonization of the supplemented species in the GI tract (1).

Studies on RA using animal models show oral treatment with probiotics decreases arthritic severity through reduced gut permeability (5, 6). The oral administration of the probiotic *Lactobacillus rhamnosus* in an *in vivo* model in Lewis rats has revealed the clinical and histopathological progresses in inflamed joints (3). A recent randomized double-blind clinical study has evaluated the effects of the oral administration of probiotic *L. rhamnosus GR-1* and *Lactobacillus reuteri RC-14* capsules on RA patients. The study demonstrated functional improvement in the probiotic group compared to the placebo group (4). Recently, the FDA granted self-affirmed status (GRAS) to a probiotic strain of *Bacillus coagulans* that can withstand the low pH of stomach acid and is activated by bile salts in the intestine and modulate the gut microbiota. *B. coagulans* GBI-30, 6086 has been used for human consumption to ameliorate the symptoms of various GI disorders and as an immuno-modulating agent for an *in vivo* human immunodeficiency virus research (7, 8).

A pilot study by Mandel et al. demonstrated the effectiveness of *B. coagulans* on RA symptoms using clinical examination and laboratory tests for erythrocyte sedimentation rate and C-reactive protein. They also suggested larger trials, given their low study population size, to verify their results (2).

To the best of our knowledge, there is a lack of information about the effects of *B. coagulans* and inulin on the progression of RA using serological markers. Our previous findings indicated significant improvement in GI microbiota, following the administration of probiotic *B. coagulans* and prebiotic inulin (9). Consequently, we conducted this study to evaluate the *in vivo* effects of probiotic *B. coagulans* and prebiotic inulin, both in combination and separately, on progression of RA using complete Freund's adjuvant (CFA) arthritis-induced rat model.

## Materials and methods

### Production of spore suspensions

The lyophilized probiotic, *B. coagulans*, was provided by Pardis Roshd Mehregan Company, Shiraz, Iran. The bacterium was then aerobically grown in the Nutrient Yeast extract Salt Medium (NYSM) agar (prepared by the method of Russell et al. (10)) at 37°C for 24 h. A single colony from the culture plate was transferred to the NYSM broth, then shaken (250 rpm) and incubated at 37°C for 48 h. The bacterial sediment was prepared by centrifugation (3,000×g for 20 min) of the bacterial suspension, which was then washed and re-suspended in 100 ml of the sterile normal saline. A heat shock at 80°C for 15 min was then applied to kill the vegetative forms of the bacteria. The spore suspension was then serially diluted and sub-cultured onto the NYSM agar. A working solution containing 1×10^9^ spore ml^−1^ was finally stored in the refrigerating temperature until further use.

### Animals and diets

Forty-eight male Wistar rats (200±8.4 g) were purchased from the Animal Centre of Razi Research Institute, Shiraz, Iran. The animals were housed in a temperature-controlled condition (22±2°C) with 55±10% relative humidity and 12-h light intervals. All the animals were subsequently categorized into six treatment groups (*n=*8) (Table 1).

**Table 1:** Treatment groups used in the experimental study

		Treatment	
		
Treatment groups	Induction of rheumatoid arthritis	Feeding	Gavaging (1 ml volume, once daily)^a^
Control	−	Standard diet	Normal saline
RA (disease control)	+	Standard diet	Normal saline
INDO (treatment control)	+	Standard diet	Indomethacin 3 mg/kg
PRE (prebiotic)	+	Standard diet+5% inulin^b^	Normal saline
PRO (probiotic)	+	Standard diet	10^9^ spores of *B. coagulans*
SYN (synbiotic)	+	Standard diet+5% inulin^a^	10^9^ spores of *B. coagulans*

a To assimilate the experimental conditions and ensure that all rats receive the total amount of prepared suspension, gavaging method was preferred.

b Long-chain inulin (Sensus, Roosendaal, The Netherlands).

The standard pellet contained 14.5% protein, 4.7% ash, 51.2% starch, 4.3% sugar, and 4% fat (3.2 kcal/g). Regarding micronutrients, the feedstuff contained 0.72% calcium, 0.6% phosphorus, 0.23% magnesium, and 0.25% chloride, among others. Levels of inulin in the diet were calculated according to the food intake. Food and distilled water were provided *ad libitum*.

### Experimental procedures

All the animals were adapted for 2 weeks before the onset of experiments. Animals in each group were fed on the diets on day 0, 2 weeks prior to the induction of RA, which were subsequently continued to the end of trials. Fourteen days following the start of experiments, the RA group was induced by the subcutaneous injection of 0.1-ml CFA (Sigma Chemicals, Saint Louis, MO, USA), containing 10 mg of heat-killed *Mycobacterium tuberculosis* per ml of paraffin oil into the plantar surface of the left hind paw (11). An equal volume of saline was injected in the control group. All the procedures were performed according to the ethical guidelines of animal welfare approved by Shiraz University and the animal welfare laws, guidelines, and policies in Iran.

### Sample collection

**Fig. 1:**
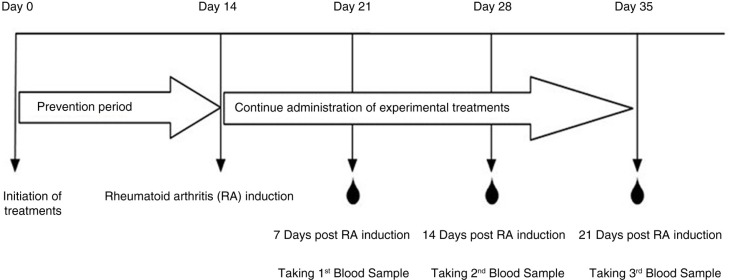
The schematic overview of the experimental study.

Animals were anesthetized using diethyl ether on day 21, 28 and 35 of trial (7, 14 and 21 days post RA induction) as it is shown in Fig. 1. Approximately 3-ml blood was directly collected by heart puncture and transferred into sterile tubes containing EDTA in order to collect plasma, which was then stored at −70°C until further use.

### Measurement of fibrinogen

The thrombin clot time (TCT) was employed to record the fibrinogen (Fn) values (Coagulometer MAC 51–52, Guangzhou Maya Equipment Co., Ltd. Guangdong, China).

### Determination of serum amyloid A

A solid-phase serum amyloid A (SAA) ELISA technique having sensitivity of 0.156 pg/ml was used to measure the SAA values (Rat SAA Elisa Kit, Cusabio Biotech Company, Wuhan, China).

### Measurement of TNF-α and alpha-1-acid glycoprotein

TNF-α and alpha-1-acid glycoprotein (α_1_*AGp*) were, respectively, measured by a solid-phase sandwich-ELISA method (AbC 607; Votre Fournisseur AbCys S.A., Paris, France) and the quantitative competitive enzyme immunoassay technique, each based on a polyclonal antibody detection system (Assaypro, MO, USA).

### Measurement of paw thickness

Arthritic inflammation was evaluated by the dorsoventral thickness of each left paw (12). Briefly, a caliper was placed at the border of the phalanges and metatarsals regions. The values were expressed as mean±SD of paw thickness.

### Statistical analysis

An ANOVA test was employed to determine the differences among the values, followed by Duncan's multiple comparison test to differentiate the mean values using SPSS (version 19, SPSS Inc.) at a significance level of 0.05.

## Results

In order to study the preventive effect of different diets on inflammatory markers, we determined plasma Fn, SAA, and TNF-α and α_1_*AGp* levels at 7, 14, and 21 days post-RA induction in rats. Details of the key events associated with the CFA-induced arthritis model are presented in Fig. 1.

**Fig. 2:**
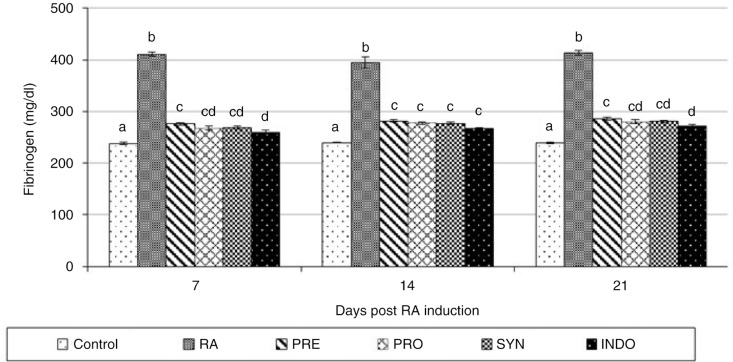
Effect of oral administration of *Bacillus coagulans* and inulin on plasma fibrinogen levels in complete Freund's adjuvant (CFA) induced male Wistar rats. For details of the treatment groups, see Table 1. Values are expressed as mean for eight animals. Bars represent standard deviation values. The different letters in the same sampling day indicate significant differences (*P*<0.05).

Figure 2 represents the Fn changes associated with arthritic conditions. As expected, the levels of Fn were increased significantly in arthritic rats (*P* < 0.001). These changes were shown to near-normal levels in PRE-, PRO-, SYN-, and INDO-treated animals.

**Fig. 3:**
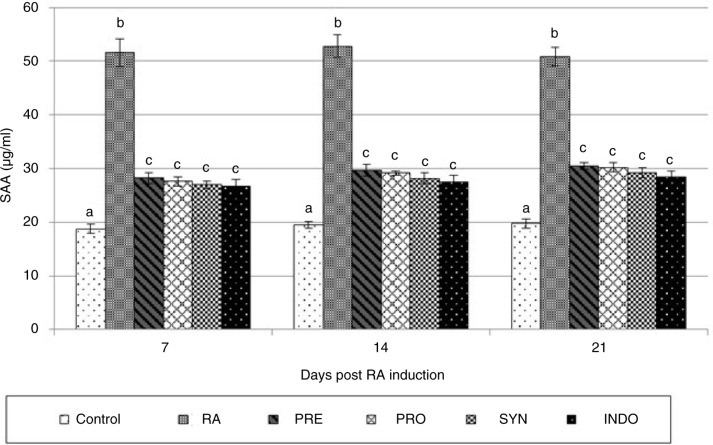
Effect of oral administration of *Bacillus coagulans* and inulin on plasma serum amyloid A (SAA) levels in CFA-induced male Wistar rats. For details of the treatment groups, see Table 1. Values are expressed as mean for eight animals. Bars represent standard deviation values. The different letters in the same sampling day indicate significant differences (*P*<0.05).

The effect of experimental diets on the SAA levels in RA-induced rats is shown in Fig. 3. By day 21, the baseline values for SAA in plasma were 19.75 µg/ml in the control rats, 50.8 µg/ml in RA, 30.5 µg/ml in the PRE, 30.2 µg/ml in the PRO, 29.2 µg/ml in the SYN, and 28.5 µg/ml in the INDO-treated rats. Overall, in all sampling days, control rats showed normal values for SAA. Surprisingly, a substantial increase in the level of SAA was shown in the arthritis-induced animals (*P* < 0.001). Results showed that PRE, PRO, and SYN diets significantly inhibited SAA production in arthritic rat models (*P* < 0.001) that was similar to indomethacin effect in drug control rats (INDO) (*P* > 0.05).

**Fig. 4:**
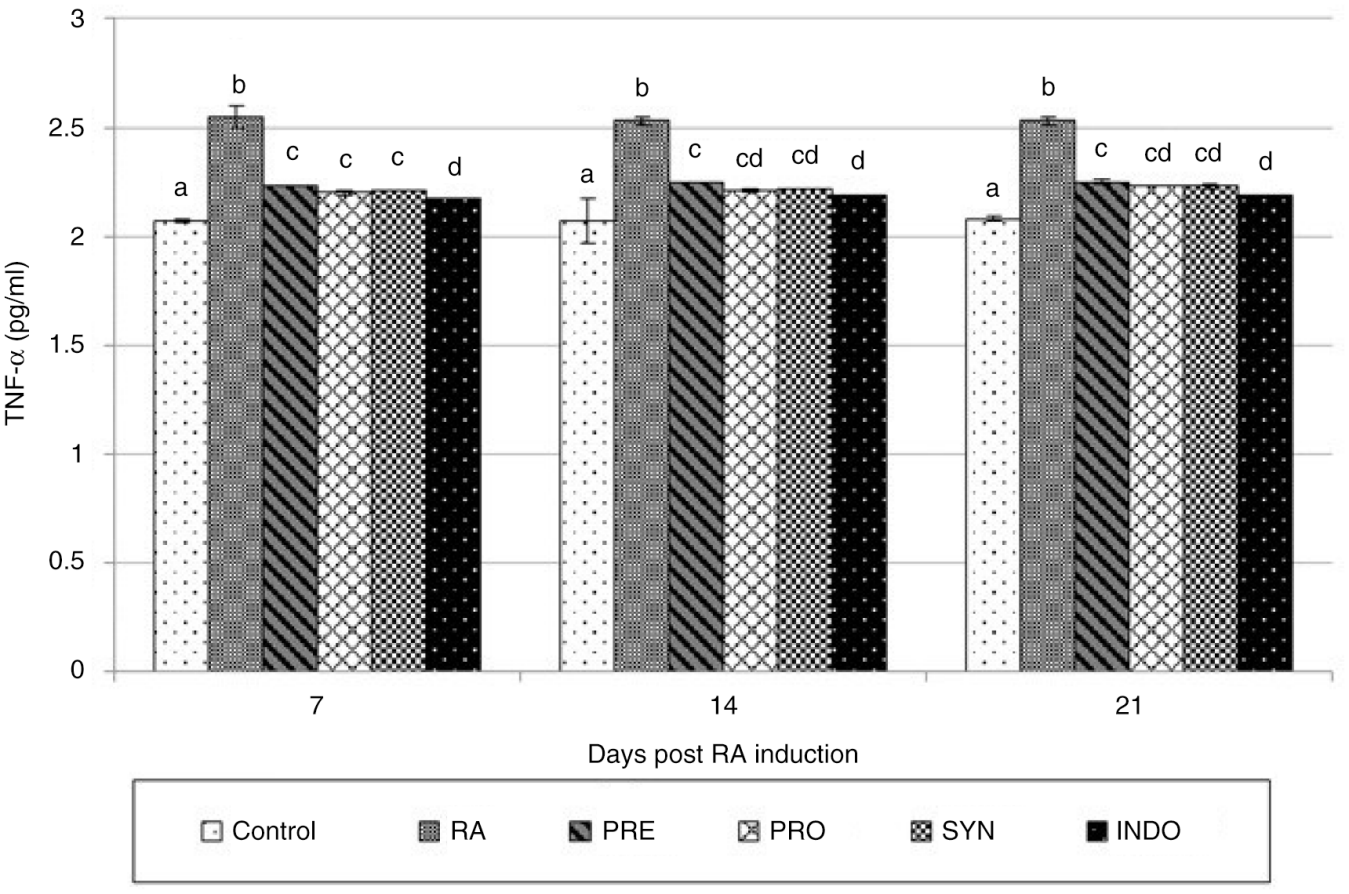
Effect of oral administration of *Bacillus coagulans* and inulin on TNF-α levels in CFA-induced male Wistar rats. For details of the treatment groups, see Table 1. Values are expressed as mean for eight animals. Bars represent standard deviation values. The different letters in the same sampling day indicate significant differences (*P*<0.05).

The subplantar injection of CFA in rat model led to a significant increase in the concentration of pro-inflammatory cytokine, TNF-α, in RA rats compared to levels in the control group (*P* < 0.001), as shown in Fig. 4, which reached to 2.55 pg/ml 1 week after RA induction and remained elevated to the end of trial. Interestingly, pretreatment with PRE, PRO, and SYN diets for 14 days before and after RA induction significantly inhibited the production of TNF-α (*P* < 0.001), which was compatible with the anti-inflammatory effects of indomethacin.

**Fig. 5:**
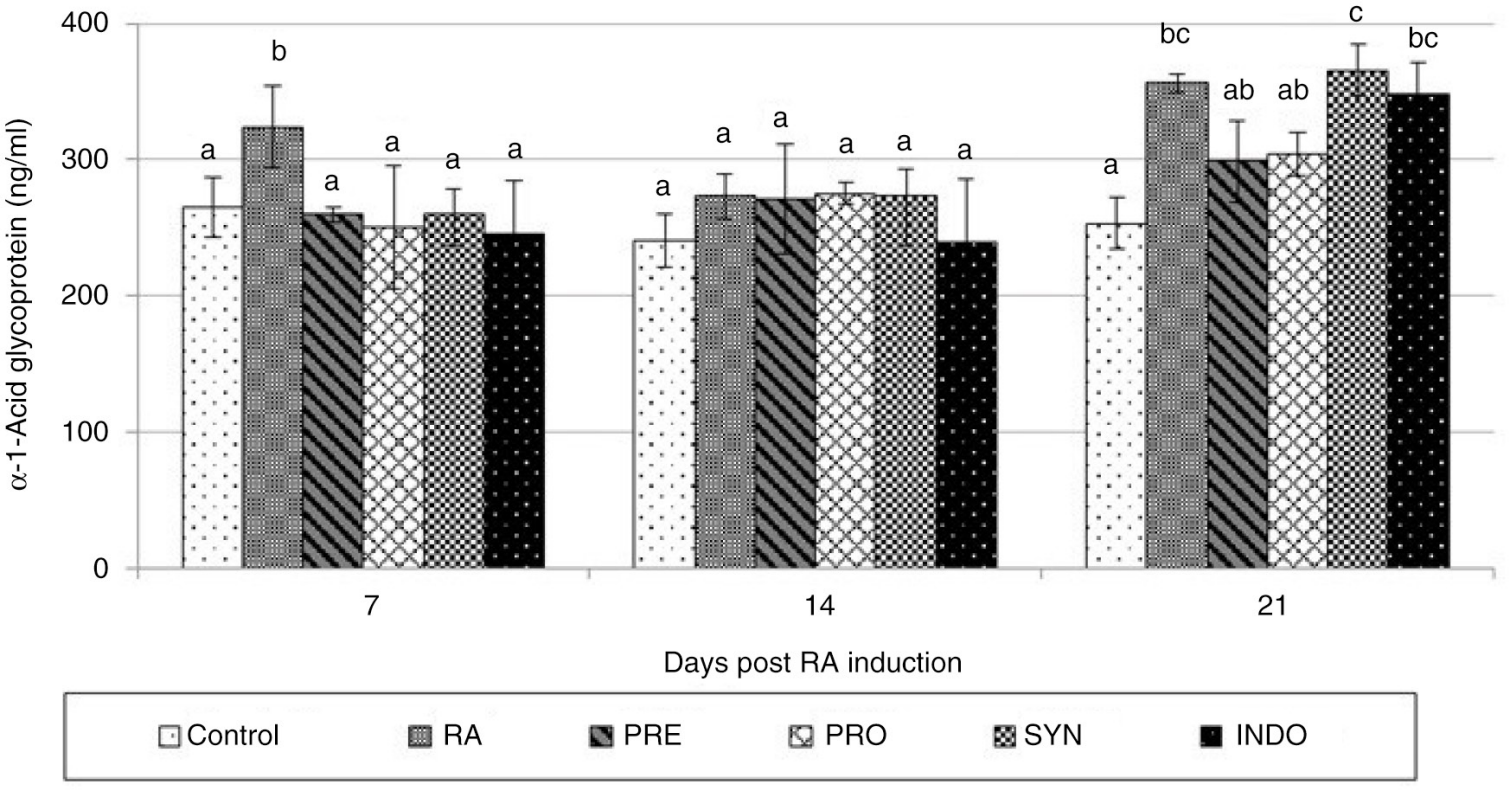
Effect of oral administration of *Bacillus coagulans* and inulin on alpha-1-acid glycoprotein (α_1_*AGp*) levels in CFA-induced male Wistar rats. For details of the treatment groups, see Table 1. Values are expressed as mean for eight animals. Bars represent standard deviation values. The different letters in the same sampling day indicate significant differences (*P*<0.05).

Figure 5 shows the preventive effect of PRE, PRO, and SYN diets on the levels of α_1_*AGp*. A significant increase was observed in the levels of α_1_*AGp* in RA animals when compared to those of the control rats (*P=*0.05). These changes were returned back to the approximate normal levels on PRE, PRO, and SYN administration, 7 days post-RA induction. On day 14, the values of α_1_*AGp* in anti-inflammatory drug rats were near normal. Further, no significant anti-inflammatory effects were observed following different treatments using α_1_*AGp* as an RA indicator.

**Fig. 6:**
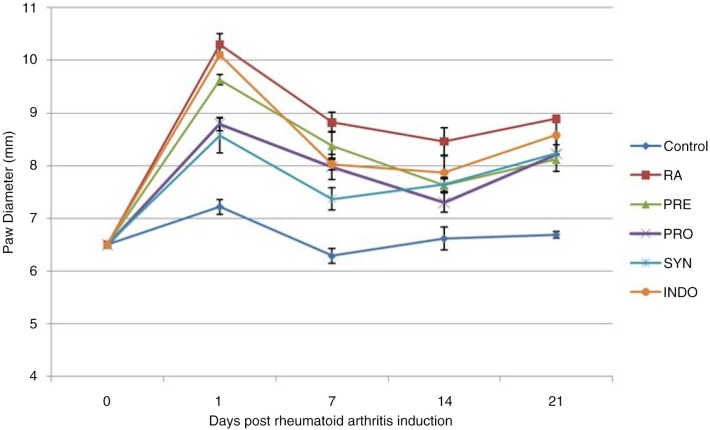
Effect of oral administration of *Bacillus coagulans* and inulin on paw diameter in CFA-induced male Wistar rats. For details of the treatment groups, see Table 1. Values are expressed as mean for eight animals. Bars represent standard deviation values.

It was obvious that the subplantar injection of CFA in all administration groups led to an increase in the paw size, as shown in Fig. 6, which reached a maximum level in 24 h. In the control group, an increase in paw diameter was observed in the first 24 h of induction, but not a significant increase (*P* > 0.05). No inhibitory effect of indomethacin was observed in the first 24 h of disease induction (*P=*0.136), while the administration of PRO and SYN had a significant preventive effect on paw inflammation (*P* < 0.001). Seven days from RA induction, the anti-inflammatory effects of PRO and SYN were similar to the indomethacin effects (*P=*0.061). Pretreatment with *B. coagulans* and inulin, both in combination and separately, 14 days after RA induction, significantly inhibited the development of the paw swelling induced by CFA (*P* < 0.001). A vigorous preventive effect against RA was observed in SYN-fed rats, 7 and 14 days post-RA induction.

## Discussion

Studies hypothesized that the disturbance in intestinal microbiota and the colonization of opportunistic bacteria in RA patients may participate in the etiopathogenesis of the disease. When intestinal dysbiosis occurs, pro-inflammatory T cells (T helper 1 (TH1) and TH17 cells) are activated by different molecules, such as SAA, and cause B cells differentiate to autoantibody-producing plasma cells. These cells and antibodies migrate to synovial tissues, which then tend to activate macrophages and the release of pro-inflammatory cytokines such as interleukin-1 and TNF-α. These stimulate synovial fibroblasts and chondrocytes in the articular cartilage, releasing the degrading proteoglycan and collagenase which finally leads to tissue destruction (1, 13). The current phenomenon has led researchers to evaluate the efficacy of probiotics and prebiotics as preventive or therapeutic interferences for relieving RA symptoms through adjusting GI tract function and reregulating local inflammatory processes (14). The CFA-induced arthritis is a T-cell-dependent model, which is characterized by reliable, rapid, and easily measurable clinical signs of arthritis (11).

Evidences from a review (15) involving modes of action of Freund's adjuvants suggested that primary target cells for the adjuvant components are mononuclear phagocytes and dendritic cells (DCs), which can activate pro-inflammatory TH17 and TH1 cells and stimulate them to produce inflammatory cytokines such as TNF-α. Secretion of these cytikines lead synovial fibroblasts and chondrocytes start joints damages, similar to RA pathogenesis in human.

Studies suggest that SAA and TNF-α play important roles as mediators of leukocyte recruitment, angiogenesis, and matrix degradation, which ultimately lead to synovial invasion and joint damage in RA (16, 17). So, the inhibition of SAA and TNF-α may be an approach to suppress and prevent inflammation causing RA either in the human disease condition or the animal model.

In this study, the effects of *B. coagulans* in PRO and SYN groups on the inhibition of TNF-α are similar to that of indomethacin. Inulin also showed a significant inhibitory effect on TNF-α release, but it was not as effective as indomethacin. One of the most extensively studied mechanisms for the immune regulatory role of probiotics is the interaction between probiotics and DCs that causes DCs maturation. It was documented that DCs have directly taken the sample antigens, such as enteropathogenic or commensal bacteria, in the intestinal lumen via transepithelial dendrites. DCs have the ability to direct T cells to assume regulatory functions through producing anti-inflammatory and regulatory cytokines, such as IL-12, IL-10, and TGF-ß (18).

The results of present study indicated that the highest SAA concentrations occurred in RA rats (50.8–52.5 µg/ml) compared with the other groups (*P* < 0.001). As shown in Fig. 3, pretreatment with PRE, PRO, and SYN diets significantly inhibits SAA production in arthritic rats. There were no significant differences between SAA values in preventive diets and the drug control group (INDO).

On the other hand, α_1_*AGp* change is associated with arthritic conditions. Although α_1_*AGp* levels were lower in the PRE, PRO, and SYN groups, they were not significant, 7 and 14 days post-RA induction (*P=*0.5, *P=*0.89). On day 21, different values of α_1_*AGp* were observed. This result is supported by Mullan et al. (17), who explained that the serum levels of some acute-phase proteins were rapidly and precisely increased when compared to those of other serum proteins. They mentioned that mild inflammation may, thus, cause increases in SAA, while not affecting proteins such as α_1_*AGp* and haptoglobin. They also found that SAA is a more sensitive acute-phase reactant in RA than a C-reactive protein, which may allow monitoring of disease activity in those patients whose other acute-phase proteins are normal (17). In another study, Cunnane et al. (19) indicated that SAA was closely associated with markers of disease activity and reflects alterations in disease status among patients with recent onset arthritis.

In this study, the injection of CFA caused severe arthritis that correlates with high levels of plasma Fn. The protein fibrinogen has an essential role in blood clotting, and it is one of the most important autoantigens in RA. Connolly et al. (20) indicated that, although Fn is synthesized by liver, it is probably produced by the inflamed joints. Systemic vasculitis, characteristic of severe arthritis in the rat model, may contribute to the high concentration of plasma Fn produced in inflamed tissue (20). Our data show that administration of PRE, PRO, and SYN to arthritic rats reduced Fn production similarly to that observed in the INDO-treated group, indicating the clear anti-inflammatory potential role of diet manipulation by *B. coagulans* and inulin.

An obvious increase in paw thickness in the first 24 h of injection in the control and treatment groups is the result of the acute inflammation that follows injection of normal saline and FCA, respectively. After the peak in paw thickness of rats following acute inflammation, a decrease correlated to immune responses was seen in all groups. Our results indicate that rats treated with *B. coagulans* either PRO or SYN groups showed significantly lesser paw thickness, similar to that of the indomethacin-administered rats. Indomethacin is a nonsteroidal anti-inflammatory drug (NSAID) that reduces pain and inflammation by blocking the enzymes that make prostaglandins (cyclooxygenase 1 and 2) and thereby reducing the paw thickness. Prostaglandins, the main cause of acute inflammation, are downregulated by anti-inflammatory cytokines. In the present study, *B. coagulans* might cause the production of anti-inflammatory cytokines that inhibit paw swelling by lowering prostaglandins production (21). In addition, the mild increase, over time, in the paw thickness of rats either in control or treatment groups is related to their body growth and weight gain.

The effects of various strains of LAB probiotics on the clinical manifestations have been, formerly investigated in the pre-clinical studies. These studies documented that probiotics, *Lactobacillus GG*, *L. casei*, *L. fermentum*, and *L. delbrueckii*, had anti-RA effects (3, 21–24).

Even though most LAB probiotic bacteria are inactivated by bile and low gastric pH, they can tolerate the unfavorable conditions of the GI as a result of the presence of a thick layer around the spores of *B. coagulans*. Bacteriocins, lactic acid, and short-chain fatty acids, such as the butyric acid produced by activated *B. coagulans*, considerably limit any opportunistic microorganisms that may contribute to an inflammatory response.

## Conclusions

The results of this study confirm that the oral intake of probiotic *B. coagulans* and prebiotic inulin can modulate the immune system by enhancing or suppressing the immune response and lessen the symptoms of induced RA in rats. This finding has important implications when planning to use functional foods, such as probiotics and prebiotics, as dietary intervention strategies in patients with RA. Further clinical studies are warranted to validate our results.

## Declaration

Part of this work was previously presented as abstract in The World Academy of Science, Engineering and Technology Conferences, San Francisco, USA, August 2015.
